# Association between serum uric acid level and non-alcoholic fatty liver disease in Koreans

**DOI:** 10.2478/abm-2022-0003

**Published:** 2022-02-28

**Authors:** Huiyul Park, Kye-Yeung Park, Minki Kim, Hoon-Ki Park, Hwan-Sik Hwang

**Affiliations:** Department of Family Medicine, Hanyang University College of Medicine, Seoul 133-791, Korea

**Keywords:** gastroenterology, hepatic fibrosis, hepatic steatosis, non-alcoholic fatty liver disease, serum uric acid

## Abstract

**Background:**

The association between serum uric acid (SUA) levels and non-alcoholic fatty liver disease (NAFLD) is controversial.

**Objectives:**

We compared the association of SUA levels with NAFLD, abnormal alanine transferase (ALT), and the degree of liver fibrosis to clarify the association of SUA levels with NAFLD.

**Methods:**

We conducted a retrospective cross-sectional study. Adult patients who underwent a health check-up (N = 1,343) were included for analysis. Fatty liver was diagnosed by abdominal ultrasonography. The degree of liver fibrosis was determined using the NAFLD fibrosis score (NFS). Pearson correlation analysis showed a stronger correlation of SUA level with the fatty liver index (*r* = 0.40, *P* < 0.001) than the correlation with serum ALT level (*r* = 0.28, *P* < 0.001), or NFS (*r* = 0.018, *P* = 0.51). SUA levels in patients with NAFLD and an abnormal liver function test (LFT) result were significantly higher than levels in patients without NAFLD and abnormal LFT results. By contrast, there was no significant association of SUA level with NFS grade. When age, male sex, body mass index, the presence of hypertension, diabetic mellitus, and NAFLD, abnormality of ALT level, and SUA level were included in binary logistic regression to evaluate risk factors for elevated NFS grade, hyperuricemia was not significantly associated with NFS grade (OR = 0.94, *P* = 0.75).

**Conclusion:**

Pearson correlation and logistic regression together indicated SUA level is more closely associated with hepatic steatosis than abnormal liver function test or hepatic fibrosis.

Non-alcoholic fatty liver disease (NAFLD) is a condition in which excess fat accumulates in the liver from causes other than alcohol. As the number of patients with obesity and metabolic diseases increases, the prevalence of NAFLD, considered to be a major cause of chronic liver disease worldwide [1, 2], has increased to 28% of the Korean population [3, 4].

NAFLD includes non-alcoholic fatty liver (NAFL) and non-alcoholic steatohepatitis (NASH). While NAFL may be associated with increased cardiovascular disease and malignancy, it is considered benign with a good prognosis from a hepatological perspective. By contrast, NASH is progressive and causes NAFLD-related cirrhosis and hepatocellular carcinoma. As NAFLD has a substantially different prognosis, it is clinically important to confirm the presence or progression of hepatic fibrosis in patients with NAFLD [3, 4]. However, there are some reports of association between serum uric acid (SUA) levels and NAFLD without consideration of the severity of NAFLD, such as the presence of NASH or hepatic fibrosis [5, 6].

Uric acid (UA), a major product of purine metabolism involved in gouty arthritis, is closely related to metabolic disorders and insulin resistance, which are the major features of NAFLD [7]. Previous studies have shown that hyperuricemia is an independent risk factor for NAFLD [8]. However, some studies suggest that NAFLD may cause hyperuricemia [9]. Although it is difficult to determine a causal relationship because of their complex interactions, it is clear that there is a strong association between NAFLD and hyperuricemia.

Similarly, when evaluating a usefulness of biomarker for NAFLD, it is crucial to confirm whether it simply indicates just the amount of fat in the liver or includes information regarding liver damage and degree of fibrosis. There have been several reports that SUA level is associated with not only hepatic steatosis, but also liver damage [10] or histological changes in the liver [11]. However, there are few studies directly comparing the relation of hyperuricemia with the amount of fat in the liver or the degree of liver fibrosis. Moreover, there is inconsistency in the association between SUA level and significant liver fibrosis [10, 12].

In the present study, we compared the associations of SUA level with the presence of NAFLD defined by abdominal ultrasound and hepatic fibrosis estimated by liver fibrosis formulas in an average risk Korean population to clarify the association of SUA level with NAFLD.

## Methods

### Study design

We conducted a retrospective cross-sectional study. The medical records of patients who had undergone a health checkup at Hanyang University Medical Center were reviewed for analysis. The Institutional Review Board (IRB) of Hanyang University Medical Center approved this study protocol (IRB file No. HYUH 2020-02-010-001, approval date 2020 Oct 20) in accordance with ICH Good Clinical Practice (GCP) Guidelines, the principles outlined in the contemporary revision of the Declaration of Helsinki (64th WMA General Assembly, Fortaleza, Brazil, October 2013), the study complies with domestic and international related laws, such as the enforcement ordinance of the Bioethics and Safety Act. Informed consent was specifically waived because of the retrospective nature of our study. Reporting is in accordance with STROBE statement [13].

### Study population

**Figure 1:**
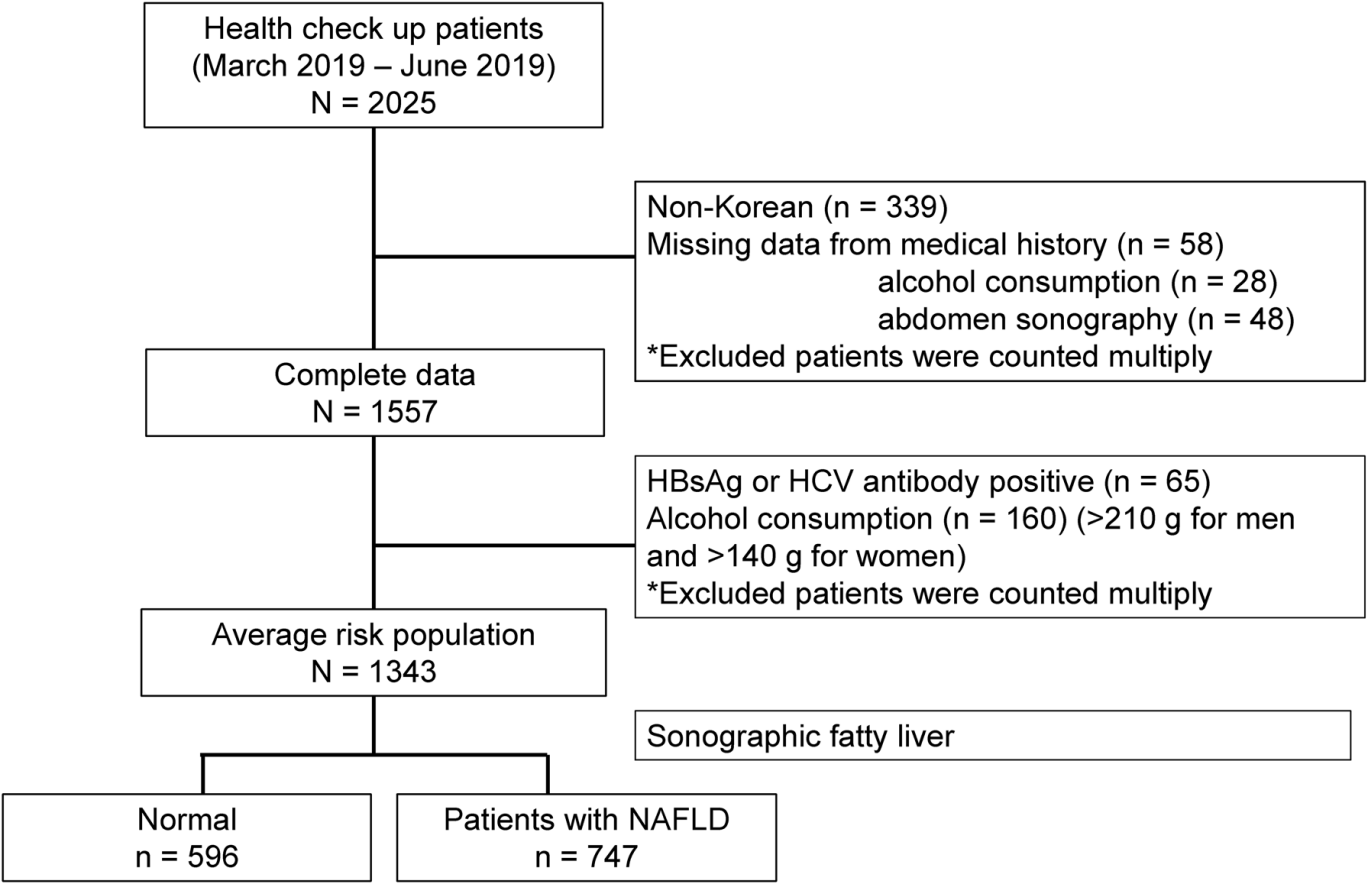
Study flow diagram. HbsAg, surface antigen hepatitis B virus; HCV, hepatitis C virus; NAFLD, non-alcoholic fatty liver disease.

In the present study, the medical records of a total of 2,025 of adult patients who had undergone health check-up at Hanyang University Medical Center between March 2019 and June 2019 were initially reviewed. We excluded data of non-Koreans (n = 339) and patients with missing data for their medical history (n = 58), abdominal ultrasonography (n = 48), or alcohol consumption (n = 28). We further excluded data of patients with a high risk of liver disease, who had the positive serological markers for hepatitis B virus (HBV) or hepatitis C virus (HCV) or who self-reported as being an HBV carrier (n = 65), as well as patients whose weekly alcohol consumption was >210 g for men or >140 g for women (n = 160). Ultimately, data of 1,343 patients were included in this study (**Figure 1**).

### Fatty liver index

Fatty Liver Index (FLI) was calculated from descriptive statistical analysis by using the equation:

(e^y^/(1 + e^y^) × 100, where y = 0.953 × ln(Tg [mg/dL]) + 0.139 × BMI [kg/m^2^] + 0.718 × ln(GGT [U/L]) + 0.053 × WC [cm] − 15.745 [14],

where Tg is triglycerides, BMI is body mass index, GGT is γ-glutamyl transferase, and WC is waist circumference.

### NAFLD fibrosis score

NAFLD fibrosis score (NFS) was calculated according to Hagström et al. [15]. NFS grades were divided into grade 0 (low risk), 1 (intermediate risk), and 2 (high risk) by using a cut-offs at −1.455 and 0.675. When age of patients was >65 years, a cut-off at 0.12 was used as the low cut-off instead of −1.455 [16].

### NAFLD, abnormal alanine transferase, and hyperuricemia

NAFLD was diagnosed by ultrasonography of Korean patients without excessive alcohol consumption or viral or genetic liver disease. The degree of fatty liver was graded as normal, mild, moderate, or severe on the degree of fat infiltration [17]. The degree of fat infiltration was evaluated by the following ultrasound parameters, such as liver echotexture, liver attenuation, and visualization of intrahepatic vessel borders or diaphragm.

Abnormal alanine transferase (ALT) was defined as having a serum ALT level ≥40 IU/L. Hyperuricemia was defined as having an SUA level of >7.0 mg/dL in men and >5.7 mg/dL in women.

### Statistical analysis

Continuous and categorical variables are presented as mean (standard deviation, (SD)) and number (percent), respectively. To compare the mean values between the groups, a Student *t* test or analysis of variance (ANOVA) were used for continuous variables. Categorical variables were compared using χ^2^ tests. The control group was defined as patients without sonographic fatty liver. Pearson correlation analysis was conducted to compare associations of SUA level with the FLI, serum ALT, and NFS. To evaluate risk factors for patients with elevated NFS grade, logistic regression was used. Age, male sex, body mass index (BMI), the presence of hypertension, diabetic mellitus (DM), and NAFLD, abnormality of ALT, and SUA levels, which were confirmed as significant risk factors in monovariate analysis for elevated NFS grade or major variables, were included. Before inclusion of the variables, variance inflation factors (VIFs) were calculated, and all VIFs were <2. Backward selection (likelihood ratio) method was used in regression analysis for selecting variables. No data of any patient with missing data was used. For all analyses, *P* < 0.05 was considered significant. Statistical analyses were conducted using IBM SPSS Statistics for Windows (version 26).

## Results

### Patient characteristics

We included data of 1,343 patients for analysis, but not that of 952 patients with risk factors for chronic liver disease or insufficient data (**Figure 1**). The baseline characteristics, demographic data, and the prevalence of hyperuricemia and metabolic disease according to presence of NAFLD are presented in **Table 1**. The mean age of the patients was 46.8 years, and the proportion of men was 57.6% (774/1,343). The prevalence of NAFLD and hyperuricemia were 55.6% (747/1,343) and 19.1% (257/1,343), respectively. Age, BMI, blood pressure, cholesterol, triglyceride, fasting blood glucose, liver enzymes, NFS, and prevalence of hyperuricemia and metabolic disease such as metabolic syndrome (MetS), hypertension, and DM in patients with NAFLD were higher than in the control group, as shown in **Table 1** (all *P* < 0.001). These values are increased with severity of hepatic steatosis by ultrasonography, as shown **Table 1** (all *P* < 0.001).

**Table 1:** Baseline characteristics, demographic data, and hyperuricemia and metabolic disease prevalence of patients according to presence of NAFLD

**Health check-up patients (N = 1,343)**	**Normal patients** **n = 596 (44.4)**	**Patients with NAFLD** **n = 747 (55.6)**	**Hepatic steatosis status**			***P* †**	***P* for trend**

			**Mild FL** **n = 435 (32.4)**	**Moderate FL** **n = 248 (18.5)**	**Severe FL** **n = 64 (4.7)**		
Age (years)	44.1 (13.0)	48.9 (10.7)	48.7 (11.2)	49.5 (10.0)	46.8 (10.0)	<0.001	<0.001
Male sex, n (%)	265 (44.5)	509 (68.1)	261 (60.0)	196 (79.0)	52 (81.3)	<0.001	<0.001
Normal weight (BMI <23)	425 (71.3)	183 (24.5)	151 (34.7)	30 (12.1)	2 (3.1)		
Overweight (BMI ≥23, <25)	119 (20.0)	236 (31.6)	155 (35.6)	75 (30.2)	6 (9.4)	<0.001	<0.001
Obese (BMI ≥25)	52 (8.7)	328 (43.9)	129 (29.7)	143 (57.7)	56 (87.5)		
Body weight (kg)	59 (10)	70 (11)	66 (10)	73 (10)	82 (13)	<0.001	<0.001
Height (cm)	165 (8)	167 (8)	166 (8)	168 (7)	170 (9)	<0.001	<0.001
SBP (mmHg)	115 (15)	123 (17)	120 (16)	126 (16)	132 (20)	<0.001	<0.001
WC (cm)	73 (7)	82 (8)	79 (7)	85 (6)	93 (9)	<0.001	<0.001
Platelet count (×10^4^/mL)	240 (47)	244 (51)	243 (52)	244 (50)	250 (51)	0.17	0.38
Fasting glucose (mg/dL)	91 (9)	101 (17)	98 (14)	103 (17)	112 (26)	<0.001	<0.001
Triglyceride (mg/dL)	85 (39)	144 (97)	118 (66)	174 (120)	203 (119)	<0.001	<0.001
AST (IU/L)	24 (9)	27 (13)	25 (11)	30 (13)	38 (18)	<0.001	<0.001
ALT (IU/L)	19 (9)	29 (23)	22 (15)	35 (27)	54 (28)	<0.001	<0.001
Total cholesterol (mg/dL)	190 (33)	199 (37)	196 (35)	203 (38)	206 (38)	<0.001	<0.001
HDL cholesterol (mg/dL)	60 (12)	52 (10)	54 (10)	49 (9)	47 (8)	<0.001	<0.001
LDL cholesterol (mg/dL)	111 (24)	122 (27)	118 (26)	126 (29)	128 (29)	<0.001	<0.001
SUA (mg/dL)	5.03 (1.29)	5.75 (1.38)	5.46 (1.32)	6.09 (1.32)	6.43 (1.51)	<0.001	<0.001
NFS	–2.44 (1.12)	–2.00 (1.15)	–2.01 (1.14)	–2.01 (1.17)	–1.86 (1.17)	<0.001	<0.001
Prevalence of hyperuricemia and metabolic disease n (%)							
Hyperuricemia (19.1)	76 (12.8)	181 (24.2)	71 (16.3)	80 (32.3)	30 (46.9)	<0.001	<0.001
HTN (22.9)	72 (12.1)	235 (31.5)	113 (26.0)	90 (36.3)	32 (50.0)	<0.001	<0.001
Low LDL or statin medication(24.3)	93 (15.6)	234 (31.3)	114 (26.2)	99 (39.9)	21 (32.8)	<0.001	<0.001
DM (6.4)	15 (2.5)	71 (9.5)	25 (5.7)	33 (13.3)	13 (20.3)	<0.001	<0.001

Data are presented as mean (SD) or number (percent).

† Between normal patients and patients with NAFLD. A Student *t* test or ANOVA were used for continuous variables. χ^2^ test was used for categorical variables.

ALT, alanine aminotransferase; ANOVA, analysis of variance; AST, aspartate aminotransferase; BMI, body mass index; MetS, metabolic syndrome; DBP, diastolic blood pressure; DM, diabetic mellitus; HDL, high density lipoprotein; HTN, hypertension; LDL, low-density lipoprotein; NAFLD, non-alcoholic fatty liver disease; NFS, NAFLD fibrosis score; SBP, systolic blood pressure; SUA, serum uric acid; WC, waist circumference.

### Prevalence of hyperuricemia according to disease condition

The mean value of SUA level (5.03 vs. 5.75 mg/dL; *P* < 0.001) was higher in patients with NAFLD than the control group, and SUA level increased up to 6.43 mg/dL according to the severity of fatty liver (*P* < 0.001). The prevalence of hyperuricemia was higher in patients with NAFLD (12.8% vs. 24.2%; *P* < 0.001), with abnormal ALT (17.0% vs. 34.5%; *P* < 0.001), and with hypertension (17.0% vs. 26.4%; *P* < 0.001) than control patients. However, the prevalence of hyperuricemia according to presence of liver fibrosis (NFS grade = 2) and DM were not significantly different (**Table 2**).

**Table 2:** Prevalence of hyperuricemia according to underlying disease

**Underlying disease condition**	**Prevalence of hyperuricemia**	***P***
NAFLD (−)	76 (12.8)	<0.001
NAFLD (+)	181 (24.2)	
ALT abnormality (−)	200 (17)	<0.001
ALT abnormality (+)	57 (34.5)	
HTN (−)	176 (17)	<0.001
HTN (+)	81 (26.4)	
Liver fibrosis (NFS grade £ 1)	254 (19.1)	0.82
Liver fibrosis (NFS grade =2)	3 (21.4)	
Diabetes mellitus (−)	243 (19.3)	0.49
Diabetes mellitus (+)	14 (16.3)	

Data are presented as number (percent). χ^*2*^ test was used to calculate *P*. ALT, alanine aminotransferase; HTN, hypertension; NAFLD, non-alcoholic fatty liver disease; NFS, NAFLD fibrosis score.

### Correlation of SUA level with hepatic steatosis, abnormal ALT, and hepatic fibrosis

**Figure 2:**
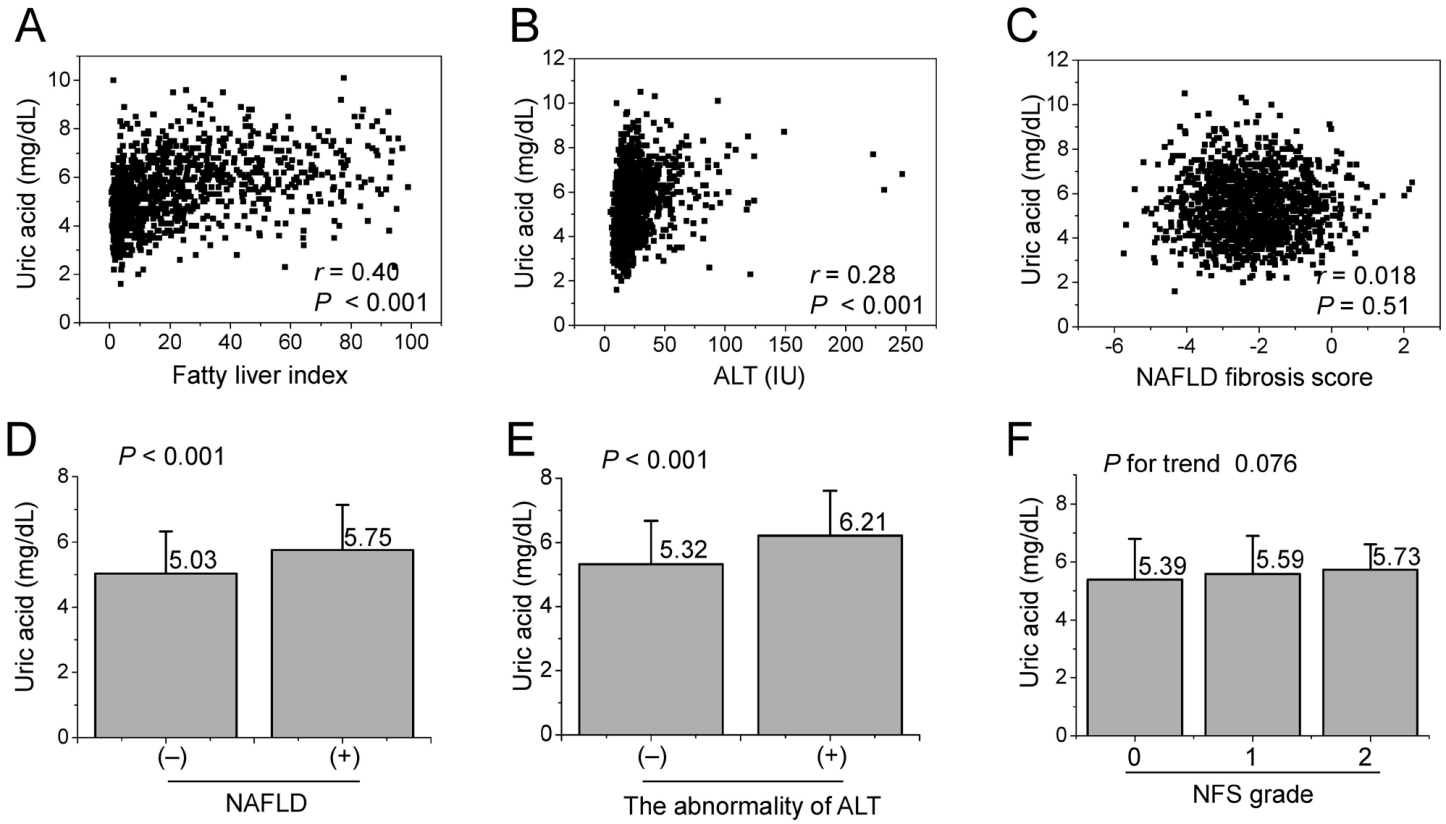
Scatter plots for SUA level versus FLI **(A)**, ALT **(B)**, and NFS **(C)**. Histograms of SUA level versus presence of NAFLD **(D)**, abnormal ALT **(E)**, and NFS grade **(F)**. Histogram bars indicate means, error bars indicate standard deviation from the mean. Pearson correlation coefficients and *P*-values are presented in the scatter plots. Abnormal ALT is defined as a serum ALT level ≥ 40 IU/L. A Student *t* test **(D, E)**, or ANOVA **(F)** was used to compare each group. NFS grade 0, advanced fibrosis was excluded; 1, needs further examination; and 2 indicates suspected advanced fibrosis. ALT, serum alanine transferase level (IU/L); ANOVA, analysis of variance; NAFLD, non-alcoholic fatty liver disease; NFS, NAFLD fibrosis score; Uric acid, serum uric acid level.

Pearson correlation analysis showed that SUA level was positively correlated with the FLI (*r* = 0.402, *P* < 0.001), ALT (*r* = 0.28, *P* < 0.001), and NFS (*r* = 0.018, *P* = 0.51) (**Figure 2A–C**). The strongest correlation was observed for SUA level with the FLI. SUA level also increased according to the presence of NAFLD and ALT abnormality (**Figure 2D and 2E**). However, there was no significant change in SUA level according to NFS grade (**Figure 2F**). When fibrosis-4 index (FIB-4) was used instead of NFS, a similar result was observed (data not shown).

### Risk assessment for elevated NFS Grade by ordinal logistic regression

To evaluate the independent association between liver fibrosis (NFS grade) and SUA level, logistic regression analysis was conducted (**Table 3**). When age, male sex, BMI, presence of hyper-tension, DM, NAFLD, and abnormality of ALT, which were confirmed as significant risk factors for elevated NFS grade in monovariate analysis, were included in logistic analysis, age, male sex, BMI, and presence of DM were significantly associated with an elevated risk of liver fibrosis. However, the presence of hypertension, NAFLD, abnormal ALT, and hyperuricemia was not significantly associated with elevated NFS grade.

**Table 3:** Multivariate risk factor analysis for patients with elevated NFS grade by logistic regression

**Variable**	**NFS grade**		**Odds ratio**	**Confidence interval**	***P***

	**0 (n = 1,051)**	**≥1 (n = 292)**			
Age (year)	44.7 (12)	54.3 (8.9)	1.08	1.06–1.09	<0.001
Male sex	572 (54.4)	202 (69.2)	1.59	1.15–2.20	0.005
BMI (kg/m^2^)	22.9 (3.1)	25.2 (3.2)	1.27	1.20–1.34	<0.001
HTN	205 (19.5)	102 (34.9)	0.73	0.51–1.04	0.085
Diabetes mellitus	36 (3.4)	50 (17.1)	2.82	1.70–4.69	<0.001
NAFLD	538 (51.2)	209 (71.6)	0.97	0.68–1.39	0.88
Abnormal ALT	115 (10.9)	50 (17.1)	0.68	0.43–1.05	0.082
Hyperuricemia	198 (18.8)	59 (20.2)	0.94	0.65–1.37	0.75

Data are presented as mean (SD) or number (percent).

ALT, alanine aminotransferase; BMI, body mass index; HTN, hypertension; NAFLD, non-alcoholic fatty liver disease; NFS, NAFLD fibrosis score.

## Discussion

To identify the association of SUA with NAFLD distinctly, we compared the associations between SUA and the presence of NAFLD, abnormal ALT, and the degree of liver fibrosis. We observed a strong association with the presence of NAFLD. However, we found no association with degree of hepatic fibrosis.

Notably, there was no significant difference in SUA and prevalence of hyperuricemia between non-DM and DM groups in our cohort, unlike the difference found by others [18]. There is a possibility of hidden medication for hyperuricemia as medication history was completely determined through self-reported questionnaires. Just 2 of 1,343 patients said they took medication for hyperuricemia, which can be omitted more easily, than medication for hypertension, diabetes, or dyslipidemia. In addition, there is a possibility that patients with DM took a statin to lower the SUA. Patients with DM showed the worse metabolic profiles such as higher blood pressure, BMI, and serum triglyceride level than patients without DM. However, total cholesterol (196 ± 34 mg/dL in non-DM vs. 171 ± 40 mg/dL in DM, *P* < 0.001) and low-density lipoprotein cholesterol level (118 ± 26 mg/dL in non-DM vs 101 ± 30 mg/dL in DM, *P* < 0.001) in patients with DM were significantly lower than levels in those without DM. The prevalence of dyslipidemia in patients with DM was 40.6% in our cohort. Dyslipidemia medication may affect SUA level of patients with DM.

Recent guidelines for NAFLD management established by the American Association for the Study of Liver Diseases (AASLD) and the Korean Association for the Study of the Liver (KASL) recommend estimating the degree of hepatic fibrosis in high-risk NAFLD patients by using liver fibrosis formulas, transient/magnetic resonance elastography, or liver biopsy because progressive hepatic fibrosis is an important surrogate for advanced liver disease. They also report that estimating fat in the liver would not be helpful for discriminating NASH from NAFLD because the quantity of fat in the liver does not match liver damage or fibrosis in NAFLD patients [19, 20]. Although SUA levels showed a mild correlation with ALT, Pearson correlation analysis showed a stronger relationship with the FLI (*r* = 0.402, FLI vs. *r* = 0.281, ALT). In addition, we found no association with fibrosis formula scores (NFS) in logistic regression (**Table 3**). So, we believe that SUA level has a stronger association with hepatic steatosis than hepatic fibrosis.

A study that included biopsy-proven NAFLD patients (n = 634) found higher levels of SUA were independently associated with hepatocellular steatosis and NASH. However, there was no association with liver fibrosis (stage F2 to F4) [21]. Recent meta-analyses by Jaruvongvanich et al. [22, 23] also found that SUA level was not associated with severity of advanced liver fibrosis (≥ F3), but liver histologic severity as determined by NAFLD activity score (NAS) in patients with NAFLD. In addition, some studies that included patients with severe liver fibrosis (liver cirrhosis) found an inverse correlation between SUA level and the degree of fibrosis because of reduced hepatic production of uric acid [24]. Our present results are consistent with previous reports suggesting that SUA is correlated not with liver fibrosis, but with the quantity of fat in the liver or abnormal ALT. The reason for not observing inverse correlation may be that our patients were otherwise generally healthy.

Hyperuricemia and NAFLD have common characteristics that include insulin resistance and oxidative stress [25]. In particular, the quantity of free fatty acids in portal venous blood is considered to drive the development of NAFLD [26]. Uric acid could drive free fatty acid to shunt into the liver and accelerate lipogenesis de novo by upregulating lipogenic enzymes or activating genes involved in lipogenesis through endoplasmic reticulum stress directly or by indirect means such as weight gain and insulin resistance [27,28,29]. Through these mechanisms, hepatic fat accumulation is stimulated by uric acid, and our finding of its high association with quantity of fat supports these explanations.

The present study has several limitations. First, the hepatic steatosis found were evaluated not by criterion standard methods such as magnetic resonance imaging (MRI) or liver biopsy, but by ultrasonography and an estimated fibrosis formula (NFS). If the data from MRI or liver biopsy was used, the results would be considered more reliable. However, routine MRI and liver biopsy measurement for diagnosing fatty liver and liver fibrosis are not possible in general health check-ups due to their high cost and invasiveness. In health check-ups ultrasonography is generally used for diagnosing fatty liver and liver biopsy is recommended to patients with a high fibrosis burden. Nevertheless, further study using data from MRI or liver biopsy will be needed to know clearly the relationship between the degree of fatty liver or fibrosis and SUA. Second, there is a possibility that data about medical and medication history might be inaccurate, because they were completely determined through self-reported questionnaires. Third, dietary information, which is highly related to NAFLD, was not considered. Fourth, the causal relationship between SUA and the main variables such as hepatic steatosis, abnormal liver enzymes, and hepatic fibrosis can not be assessed because of the observational study design. Moreover, the degree of correlation in Pearson correlation analysis was not sufficiently high to conclude clinical importance. Large-scale longitudinal studies using criterion standard methods to evaluate liver fibrosis and include clinical outcomes would be needed to provide causal evidence. The present findings for our Korean patients may not be applicable to other populations with different ancestry.

Nevertheless, a study using a biopsy-proven NAFLD cohort [15] and review article [30] to evaluate the use of noninvasive assessment for NAFLD reported NFS showed not only reasonable diagnostic performance for liver fibrosis (negative predictive value [NPV] = 88%–95% for grade 0, positive predictive value [PPV] = 75%–90% for grade 2), but also good correlation with disease severity, and the American Association for the Study of Liver Diseases (AASLD) recommends using fibrosis formulas, such as NFS or FIB-4 for patients with NAFLD at high risk to evaluate their liver fibrosis burden. Moreover, the present study was focused not on showing the diagnostic performance of SUA level for significant or advanced liver fibrosis, but what variables SUA levels correlates with. When data were reanalyzed using the FIB-4 to compensate for our limitations, similar results were obtained (data not shown). Taken all together, we consider that NFS reflects well the tendency to hepatic fibrosis in our otherwise healthy Korean patients.

## Conclusions

The strongest association of SUA levels in generally healthy Korean patients was observed with the presence of NAFLD, and there was no association with the degree of hepatic fibrosis in multivariable logistic regression. We consider that SUA level is more closely associated with the hepatic steatosis than abnormal LFT or hepatic fibrosis.
